# Updating Beliefs under Perceived Threat

**DOI:** 10.1523/JNEUROSCI.0716-18.2018

**Published:** 2018-09-05

**Authors:** Neil Garrett, Ana María González-Garzón, Lucy Foulkes, Liat Levita, Tali Sharot

**Affiliations:** ^1^Affective Brain Laboratory, Experimental Psychology, University College London, London WC1H 0AP, United Kingdom,; ^2^Department of Psychology, University of Sheffield, Sheffield S10 2TP, United Kingdom,; ^3^Princeton Neuroscience Institute, Princeton University, Princeton, New Jersey 08540, and; ^4^Department of Experimental Psychology, University of Oxford, Oxford OX2 6HG

**Keywords:** anxiety, information processing, optimism, risk, stress, threat

## Abstract

Humans are better at integrating desirable information into their beliefs than undesirable information. This asymmetry poses an evolutionary puzzle, as it can lead to an underestimation of risk and thus failure to take precautionary action. Here, we suggest a mechanism that can speak to this conundrum. In particular, we show that the bias vanishes in response to perceived threat in the environment. We report that an improvement in participants' tendency to incorporate bad news into their beliefs is associated with physiological arousal in response to threat indexed by galvanic skin response and self-reported anxiety. This pattern of results was observed in a controlled laboratory setting (Experiment I), where perceived threat was manipulated, and in firefighters on duty (Experiment II), where it naturally varied. Such flexibility in how individuals integrate information may enhance the likelihood of responding to warnings with caution in environments rife with threat, while maintaining a positivity bias otherwise, a strategy that can increase well-being.

**SIGNIFICANCE STATEMENT** The human tendency to be overly optimistic has mystified scholars and lay people for decades: How could biased beliefs have been selected over unbiased beliefs? Scholars have suggested that although the optimism bias can lead to negative outcomes, including financial collapse and war, it can also facilitate health and productivity. Here, we demonstrate that a mechanism generating the optimism bias, namely asymmetric information integration, evaporates under threat. Such flexibility could result in enhanced caution in dangerous environments while supporting an optimism bias otherwise, potentially increasing well-being.

## Introduction

Whether a piece of news is good or bad is critical in determining whether it will alter our beliefs. In particular, people readily incorporate favorable news into their existing beliefs, yet tend to underweight the strength of unfavorable information (Eil and Rao, 2011; Sharot et al., 2011; Mobius et al., 2012; Kuzmanovic et al., 2015, 2016; Wiswall and Zafar, 2015; Kuzmanovic and Rigoux, 2017; Lefebvre et al., 2017). For example, when learning that their risk of experiencing future aversive events, such as robbery, is higher than they had expected, people are less likely to integrate these data into prior beliefs relative to a situation in which they learn that their risk is lower than expected (Sharot et al., 2011). The same pattern emerges when people receive desirable and undesirable information about their financial prospects (Wiswall and Zafar, 2015) or feedback about their intellectual abilities (Eil and Rao, 2011; Mobius et al., 2012), personality (Korn et al., 2012), and physical traits (Eil and Rao, 2011). This is known as a valence-dependent learning asymmetry (Sharot and Garrett, 2016).

Incorporating desirable information about the self at a higher rate than undesirable information (Korn et al., 2012) will subsequently lead to overconfidence and optimistically biased predictions (Sharot et al., 2011). On the upside, an optimistic outlook, even when biased, can improve physical and mental health (Taylor and Brown, 1988) and boost motivation (Bandura, 1989), exploration (Tiger, 1979), and persistence (Sherman, 1980), thus enhancing success and well-being (for review, see Chang, 2001). However, ignoring negative information can result in faulty assessment and lack of precautionary action leading to, for example, ill preparedness in the face of natural disasters and financial market bubbles (Shefrin, 2009) .

These apparent costs present a conundrum: Why have humans evolved a bias in learning that leads to systematic errors in judgment? The common answer is that people make errors that are costly in certain situations because those errors are advantageous in other situations, and on balance the benefits outweigh the costs (McKay and Dennett, 2009). There is another possibility, though, that the asymmetry fluctuates in response to environmental demands. For example, in relatively safe surroundings, where potential harm is low, an asymmetry in information integration may be prominent leading to biased expectations. Yet in environments rife with threats, a physiological/psychological response may trigger changes to how information is integrated leading to more balanced information integration, which may be adaptive in environments where potential costs are high.

Because affect provides an internal signal about the external context, it could potentially be used to adaptively modulate cognitive biases. Specifically, we suggest that the key is a learning mechanism that is modulated by the two core aspects of affect: valence and arousal. A valence-dependent learning mechanism biases judgments, and an arousal-dependent switch controls the degree and perhaps sign of the bias.

**Figure 1.**
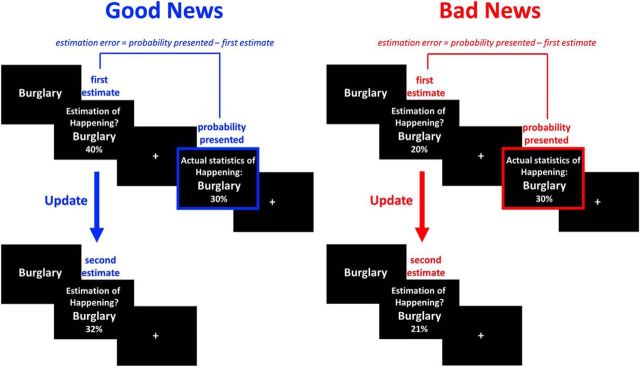
Behavioral task. On each trial, participants were presented with a short description of an adverse event and asked to estimate how likely this event was to occur to them in the future. They were then presented with the probability of that event occurring to someone from the same age, location, and socioeconomic background as them. The second session was the same as the first except that the average probability of the event to occur was not presented. Shown are examples of trials for which the participant's estimate was higher or lower than the statistical information provided leading to receipt of good news (left) and bad news (right), respectively. Note that the blue and red boxes are just for illustration and did not appear in the actual experiment.

To test this prediction, we exposed participants to an acute threat manipulation in the laboratory (Experiment I) or tested participants in a real-life environment (firefighters tested on call; Experiment II). After measuring indicators of arousal, stress, and anxiety, participants completed the belief update task (Sharot et al., 2011, 2012a,b; Moutsiana et al., 2013, 2015; Chowdhury et al., 2014; Garrett and Sharot, 2014; Garrett et al., 2014; Korn et al., 2014; Kuzmanovic et al., 2015, 2016; Kappes et al., 2018; Fig. 1). Past studies have shown that participants put more weight on good news (i.e., that a negative life event is less likely to occur than expected; Fig. 1, left) compared with bad news (i.e., that a negative event is more likely to occur than expected; Fig. 1, right) in altering beliefs in this task. Here, we test whether heightened response to threat abolishes this bias.

## Materials and Methods

### Experimental design and statistical analysis: Experiment I

#### Participants

Thirty-six participants recruited via the University College London participant pool participated in the study. Participants gave informed consent and were paid for their participation. The study was approved by the Research Ethics Committee of University College London. One participant's responses resulted in only two good news trials (of a possible 40), which prevented us from calculating a meaningful information integration parameter (we define how we calculate information integration parameters below); thus, this participant's data had to be excluded. Two participants' cortisol samples were insufficient for analysis, and samples of six participants who were suspected to have depression (Beck Depression Inventory score >10) were never sent to be analyzed. Thus, analysis that includes cortisol scores is given for *n* = 27. Each participant was randomly assigned to either the threat manipulation condition (13 females, 6 males; mean age, 26.37 years; standard deviation (SD), 6.58) or the control condition (10 females, 6 males; mean age, 24.94 years; SD, 3.82).

#### Manipulation procedure

We designed the experiment such that the perceived threat was unrelated to the information presented in the task. Thus, we could test whether the effect of perceived threat on information integration was general rather than specific to the source of the threat itself.

Participants assigned to the threat manipulation group were told that they would be exposed to an uncomfortable, stressful event at the end of the study. Specifically, they were informed that, at the end of the experiment, they would be required to deliver a speech on a surprise topic, which would be recorded on video and judged live by a panel of staff members. They were shown an adjacent room across a double mirror window where chairs and tables were already organized for the panel. In addition, participants were presented with six difficult mathematical problems that they were asked to try and solve in 30 s. This manipulation is a variation of the Trier Social Stress Test (TSST; Birkett, 2011) with the main difference between the typical TSST procedure and the one used here being that participants were threatened by the possibility of a stressful social event and completed the main task under threat, but the threat was never executed. Having the participants believe the stressful event will take place at the end of the task, rather than before, increased the likelihood that participants' arousal levels remained high throughout the task. Participants assigned to the control condition were informed that, at the end of the experiment, they would be required to write a short essay on a surprise topic, which would not be judged. They were then presented with six elementary mathematical problems to solve in 30 s.

#### Manipulation check

We examine whether the threat manipulation resulted in the following psychological and physiological changes, which are typically observed in studies using variations of TSST (Birkett, 2011).

##### Self-report.

Before and after the induction procedure, participants filled out a short form of the state scale of the Spielberger State Trait Anxiety Inventory developed by Marteau and Bekker (1992). Participants reported their current anxiety state according to six statements (e.g., I am worried) on a four-point Likert scale (1, not at all, to 4, very much). Possible scores range from 6 to 24 with high scores indicating high levels of state anxiety.

##### Skin conductance level.

Skin conductance level (SCL) is an index of sympathetic tone that reflects changes in autonomic arousal. Skin conductance was recorded for 2 min before and after induction while participants stared at a fixation cross using disposable electrodermal gel electrodes (EL507, Biopac) attached to the distal phalanx of the pointer and middle fingers of the participants' nondominant hand. Skin conductance responses were monitored using a MP36R system (BIOPAC Systems) and analyzed with BIOPAC software Acq*Knowledge*. The difference in mean SCL in each period was taken as a change in participants' autonomic arousal levels.

##### Cortisol level.

To measure changes in participants' cortisol levels, saliva samples were collected using Salivette collection devices (Salimetrics). Four samples were taken at different time points: before the induction procedure (baseline, t0), immediately after the induction procedure but before undertaking the task (10 min after the threat/control manipulation, t1), halfway through the task (30 min after the threat/control manipulation, t2), and after the task and completion of postexperiment questionnaires (+1 h after the threat/control manipulation, t3). The experiment was conducted between 2 and 4 P.M., restricted to these times to control for the diurnal cycle of cortisol. Samples were stored at −80°C before being assayed. Analysis of salivary cortisol was completed by Salimetrics. Intra-assay and interassay coefficients of variation were all below 6.1% [mean, 1.5%; SD, 1.2]. Cortisol values were measured in micrograms per deciliter. Shapiro–Wilk (SW) tests on cortisol levels at each sample period revealed that these were not normally distributed (one-sample SW < 0.01 for all four sample intervals). As a result, cortisol values were log transformed. Since cortisol stress response has a temporal delay [mediated by the slower time scale hypothalamic–pituitary–adrenal (HPA) axis], it is difficult to precisely align the time of the cortisol response to perceived levels of threat at different points in the task. Because of this, the main cortisol measure we used in the study was calculated as the mean difference between cortisol levels at time periods t1, t2, and t3 from baseline cortisol levels at t0, as done previously (Lighthall et al., 2013; Otto et al., 2013; Lenow et al., 2017). This measure represents the average cortisol response throughout the duration of task performance. Below is the formula we used to derive this index, where log cort is the natural log-transformed cortisol (cort) concentrations:

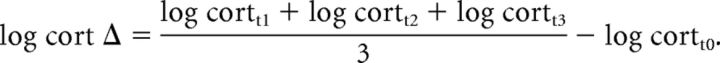


#### Behavioral task

The task was adopted from past studies (Sharot et al., 2011, 2012a,b; Moutsiana et al., 2013, 2015; Chowdhury et al., 2014; Garrett and Sharot, 2014; Garrett et al., 2014; Korn et al., 2014).

#### Stimuli

Stimuli (80 short descriptions of different negative life events, e.g., domestic burglary, card fraud) were separated into two lists, each containing 40 events. Participants were randomly assigned one of the two lists of 40 events at the start of the experiment. For each event, the average probability of that event occurring at least once to someone from the United Kingdom within the same age range as the participants was calculated from data compiled from online resources (including the Office for National Statistics and PubMed). Very rare or very common events were not included; all event probabilities lay between 10 and 70%. To ensure that the range of possible overestimation was equal to the range of possible underestimation, participants were told that the range of probabilities lay between 3 and 77%, and they were only permitted to enter estimates within this range. Note that differences between the average probabilities provided to participants and the actual probabilities for the sample of participants tested cannot explain differences between the two groups, as we randomly assign participants to either the threat manipulation condition or the control condition.

#### Behavioral task

Participants completed a practice session comprising three trials before beginning the main experiment (Fig. 1). The main experiment comprised 40 trials. On each trial, 1 of 40 adverse life events was presented for 3 s, and participants were asked to estimate how likely the event was to happen to them in the future. Participants had up to 5 s to respond. If participants had already experienced an event in their lifetime, they were instructed to estimate the likelihood of that event happening to them again in the future. If the participant failed to respond, that trial was excluded from all subsequent analyses (Mean number of missed responses, 1.31; SD, 1.39). After presentation of a fixation cross (5–10 s jittered), participants were then presented with the base rate of the event in a demographically similar population for 2 s, followed by a fixation cross (5–10 s jittered). In a second session, immediately after the first, participants were asked again to provide estimates of their likelihood of encountering the same events so that we could assess how they updated their estimate in response to the information presented.

Note that studies have shown that the update bias exists both when classifying trials according to participants' estimates of self-risk and when trials are classified according to estimates of base rates (Garrett and Sharot, 2014; Kuzmanovic et al., 2015). Thus, we used the traditional design and analysis here (Sharot et al., 2011). Moreover, multiple past studies have shown that the amount of update bias does not alter whether participants are asked to estimate the likelihood of the event happening in the future or the likelihood of the event not happening in the future (Sharot et al., 2011; Garrett and Sharot, 2014; Garrett et al., 2014). Thus, scores are not driven by response to high and low numbers but rather by valence per se. As this has been established in the past, we used the standard version of the task here (i.e., eliciting estimation of an event happening).

#### Memory control

To test for memory effects, participants were asked at the end of the experiment to provide the actual probability previously presented of each event. Memory errors were calculated as the absolute difference between the probability previously presented and the participants' recollection of that statistic: memory error = |probability presented − recollection of probability presented|.

#### Other controls

At the end of the experiment, participants also rated stimuli on six-point scales for vividness [for the question “How vividly could you imagine this event?” (1, not at all vivid, to 6, very vividly)], familiarity [for the question “Regardless if this event has happened to you before, how familiar do you feel it is to you from TV, friends, movies, and so on?” (1, not at all familiar, to 6, very familiar)], prior experience [for the question “Has this event happened to you before?” (1, never, to 6, very often)], emotional arousal [for the question “When you imagine this event, how emotionally arousing do you find the image in your mind?” (1, not at all arousing, to 6, very arousing)], and negativity [for the question “How negative would this event be/is this event for you?” (1, not negative at all, to 6, very negative)].

#### Statistical analysis

Trials were partitioned according to participants' first estimates into ones in which participants received good news (i.e., the probability presented was lower than the first estimate of their own probability; Fig. 1, left) or bad news (i.e., the probability presented was higher; Fig. 1, right). Although information can be better or worse than expected, all stimuli are negative (i.e., robbery, card fraud); thus, comparison is never between positive and negative stimuli but between information that is better or worse than expected.

Trials for which the estimation error was zero were excluded from subsequent analyses as these could not be categorized into either condition (Mean number of uncategorizable trials, 0.89; SD, 0.92).

For each trial, an estimation error term was calculated as the unsigned difference between the probability presented and participants' first estimate on that trial: estimation error = |probability presented − first estimate|.

Update was calculated for each trial such that positive updates indicate a change toward the probability presented [update (good news) = first estimate − second estimate] and negative updates indicate a change away from the probability presented [update (bad news) = second estimate − first estimate].

Formal models suggest that learning from information that disconfirms one's expectations is mediated by a prediction error signal that quantifies a difference between expectation and outcome (Sutton and Barto, 1998). We have previously shown that an analogous mechanism underpins belief updating in this task (Sharot et al., 2011). Specifically, the absolute difference between participants' initial estimations and the information provided (i.e., estimation error = |probability presented − first estimate|) predicts subsequent updates, as would be expected from learning models (Sutton and Barto, 1998). Hence, similar to our previous studies (Sharot et al., 2011; Moutsiana et al., 2013; Garrett et al., 2014), we estimated the extent to which participants integrated new information into their beliefs by correlating absolute estimation errors and update scores with one another separately for good and bad news trials for each participant. This resulted in two Pearson correlation values for each participant: one for good news trials and one for bad news trials. We denote these Pearson correlation scores as good news (α_G_) and bad news (α_B_) information integration parameters. Shapiro–Wilk tests were applied to check that the values of α_G_ and α_B_ were normally distributed. To check that the values of α_G_ and α_B_ were not at floor or ceiling, we conducted one-sample *t* tests (separately α_G_ and α_B_) against values of 0 (to test for floor effects) and 1 (to test for ceiling effects).

To determine whether information integration from good and/or bad news was altered by the threat manipulation, the resulting information integration parameters were submitted to a 2 by 2 ANOVA with valence (good/bad news) as a repeated measure and group (threat manipulation/control) as a between-subjects factor.

We identified possible confounds to add as covariates to our analysis as follows. First, for factors that were not task related and therefore did not have a valence component (specifically, initial self-reported anxiety, initial SCL, initial cortisol, and BDI), we conducted independent-sample *t* tests (control vs threat manipulation group) for each factor separately to determine whether a group difference existed (Table 1). For task-related variables that could be divided by valence (specifically, number of trials, memory scores, ratings on familiarity, vividness, past experience, negativity, emotional arousal, and mean first estimates), we calculated the difference between mean good news and mean bad news for each participant for each of these factors. This gives a bias score for each factor for each subject whereby positive scores indicate a bias toward good news and negative scores indicate a bias toward bad news. We then conducted a one-sample *t* test (vs 0) on each of these scores for each group separately to isolate those factors that had valence effects in either set of participants. Next, we conducted a series of independent-sample *t* tests to compare the control group's difference scores to the threat manipulation group's scores for each factor (this is equivalent to testing for an interaction between valence and group). For all of these tests, we applied a threshold of *p* < 0.05 and deliberately did not correct for multiple comparisons. This is because the purpose was to identify all potential confounds; by not correcting, we are being more stringent. Any factor that showed a group effect or a valence effect was added as a covariate. These were: initial self-reported anxiety, mean first estimates, ratings of vividness, familiarity, past experience, and emotional arousal (Table 1).

**Table 1.** BDI, initial self-report Spielberger State Trait Anxiety Inventory (STAI), initial SCL, initial cortisol, task-related variables, subjective scales, and memory in Experiment I

	Threat manipulation group mean (SD)	Control group mean (SD)
BDI and baseline stress levels		
BDI	5.79 (5.23)	4.69 (3.22)
Initial self-report STAI*^a^*	10.37 (2.65)	8.63 (1.36)
Initial SCL	6.27 (3.29)	5.90 (3.20)
Initial cortisol (log transformed)	−1.99 (0.59)	−1.79 (0.53)
Task variables		
First estimates	29.82 (5.62)*^b^*	31.05 (5.89)*^b^*

Subjective scales questionnaire (1, low, to 6, high)	Bias (good news–bad news)	
Vividness	0.41 (0.72)*^b^*	0.72 (0.65)*^b^*
Familiarity	0.30 (0.69)	0.49 (0.62)*^b^*
Prior experience	0.18 (0.61)	0.33 (0.41)*^b^*
Emotional arousal	0.33 (0.63)*^b^*	0.13 (0.86)
Negativity	0.20 (0.49)	−0.13 (0.58)
Other task-related variables		
Number of trials	−1.58 (8.99)	−1.56 (9.70)
Memory errors	−1.23 (3.16)	−0.21 (4.52)
Estimation errors (absolute)	−0.82 (5.27)	1.11 (5.84)
Update	2.60 (12.67)	4.21 (7.83)*^b^*

Note that estimation errors and update (the final two rows) are the variables used to compute the information integration parameters (α_G_ and α_B_) for each participant.

*^a^*Difference between the threat manipulation and control groups, tested using an independent-sample *t* test (*p* < 0.05).

*^b^*Significant effect of valence (*p* < 0.05), tested using a one-sample *t* test on the bias scores (difference between good and bad news) on each group separately.

To explore whether differences in information integration related to any of the specific physiological and psychological changes, we constructed a general linear model (GLM) with α entered as the dependent variable and changes in SCL, self-report anxiety, and cortisol as independent variables. This was done separately for information integration parameters for good (α_G_) and bad (α_B_) news. To control for general changes in information integration and allow us to detect valence-specific effects, we entered information integration parameters for good news (α_G_) as a covariate when examining information integration parameters for bad news (α_B_) and vice versa (Moutsiana et al., 2013). In addition, following the same selection procedure outlined above, we controlled for any variable where there was a significant (*p* < 0.05) difference between groups, between types of information (i.e., valence), or a group * valence interaction by including these in the GLM as covariates.

For α_B_, the formula for the regression in full is as follows: α_B_ = β0 + β1 * change in SCL + β2 * change in self-report + β3 * change in cortisol + β4 * mean initial estimate + β5 * initial self-report anxiety + β6 * mean bad news vividness rating + β7 * mean bad news familiarity rating + β8 * mean prior experience bad news rating + β9 * mean emotional arousal bad news rating + β10 * α_G_.

For α_G_, the formula for this was as follows: α_G_ = β0 + β1 * change in SCL + β2 * change in self-report + β3 * change in cortisol + β4 * mean initial estimate + β5 * initial self-report anxiety + β6 * mean good news vividness rating + β7 * mean good news familiarity rating + β8 * mean prior experience good news rating + β9 * mean emotional arousal good news rating + β10 * α_B_.

Finally, we reran the analysis above, this time controlling for within-subject covariates at the within-subject level and between-subject factors at the between-subject level. Specifically, for each participant we computed an alternative set of information integration parameters, one for good news (α_G_partial_) and one for bad news (α_B_partial_), by carrying out a series of partial correlations in which absolute estimation error and update were the two variables of interest. Within-subject covariates, identified as above (first estimate, vividness, familiarity, past experience, and emotional arousal), were controlled for on a trial-by-trial basis. We examined whether these alternative information integration parameters for bad news (α_B_partial_) related to change in self-report and/or change in SCL controlling for any additional between-subject confounds as above (initial self-report anxiety ratings and information integration for good news). This was done by entering alternative information integration parameters for bad news (α_B_partial_) as the dependent variable into two GLMs as follows: α_B_partial_ = β0 + β1 * change in self-report + β2 * initial self-report anxiety + β3 * α_G_partial_ and α_B_partial_ = β0 + β1 * change in SCL + β2 * initial self-report + β3 * α_G_partial_.

We then examined the significance of the regression weights in each GLM for change in self-report and change in SCL. To visualize the effect of each of these (see Fig. 4), we generated two partial regression plots. These are scatterplots of the residuals of the dependent variable (α_B_partial_) and the independent variable (either change in self-report or change in SCL) when these are regressed on the rest of the independent variables (initial self-report and α_G_partial_).

We ran the equivalent analysis for good news (α_G_partial_) as follows: α_G_partial_ = β0 + β1 * change in self-report + β2 * initial self-report + β3 * α_B_partial_ and α_G_partial_ = β0 + β1 * change in SCL + β2 * initial self-report + β3 * α_B_partial_.

### Experimental design and statistical analysis: Experiment II

#### Participants

Thirty-three operational staff stationed across 17 fire stations within the South Metro Fire and Rescue Authority of the State of Colorado in the United States participated in the study. Five of these participants failed to complete the study, leaving 28 participants (1 female, 27 males; mean age, 43.15 years; SD, 9.87). A link to an online version of the experiment was sent by email to operational staff inviting them to participate in the study while on duty. Employees were given 18 d to attempt the experiment. They were permitted to take the experiment once in this time period and were explicitly requested to do so while on shift (i.e., in the station between calls). Participation in the experiment was anonymous, voluntary, and unpaid.

#### Task, stimuli, and control variables

An online version of the task used in Experiment I was designed using Qualtrics Survey software (Qualtrics). The task began by asking basic demographic questions (age, gender, marital status, level of education, and number of children) and some questions pertaining to their work (including how long they had worked in the service, how many people they supervised, number of emergencies they went on, and what their rank in the service was) and social environment (social support at work and outside and stress experienced at home).

After providing this information, participants read task instructions on screen at their own pace and then undertook a practice session comprising three practice trials. As in Experiment I, stimuli (80 short descriptions of different negative life events; the majority of these were the same as those used in Experiment I, but 18 events were exchanged with alternative negative life events) were separated into two lists, each containing 40 negative life events. Participants were randomly assigned one of the two lists of 40 events at the start of the experiment. The task was the same as in Experiment I, except that there was only one fixation cross displayed in each session (for 1 s) after participants submitted estimates (i.e., in the first session, unlike in Experiment I, a second fixation cross was not displayed after base rate presentation). Furthermore, mindful of the firefighters' unpredictable time constraints, memory for the information given and subjective ratings (past experience with the event and negativity) were elicited for half the stimuli and participants completed a short version of the state scale of the self-report at the beginning of the study (Chlan et al., 2003), without providing physiological measures of autonomic arousal.

#### Statistical analysis

Linear regressions were performed using ordinary least squares implemented using SPSS version 25 for bad news and good news separately, with α entered as the dependent variable and self-reported state anxiety entered as the independent variable. To rule out potential confounds, we followed a similar procedure as in Experiment I. Specifically, we separately tested whether a range of potential confounding factors had valence effects. These factors were mean first estimates, memory scores, ratings of negativity, ratings of past experience, and number of trials. We did this by calculating the difference between mean good news and mean bad news for each participant for each of these factors. This gives a bias score for each factor for each subject whereby positive scores indicate a bias toward good news and negative scores indicate a bias toward bad news. We then conducted a one-sample *t* test (vs 0) on each of these scores to identify factors that had valence effects. We used a threshold of *p* < 0.05 and deliberately did not correct for multiple comparisons. This is because the purpose was to identify all potential confounds; by not correcting, we are being more stringent. Any factor that showed a valence effect was then added as a covariate. These were mean first estimates, ratings of past experience, and number of trials (Table 2).

**Table 2.** Task-related variables, subjective scales, and memory in Experiment II

	Mean (SD)
BDI	6.82 (7.45)
Task variables	
First estimates*^a^*	31.22 (6.96)
Subjective scales questionnaire (1, low, to 6, high)	Bias (good news–bad news)

Prior experience*^a^*	0.54 (0.94)
Negativity	0.31 (0.90)
Other task-related variables	
Number of trials*^a^*	−10.89 (9.41)
Memory errors	−2.18 (6.51)
Estimation errors (absolute)*^a^*	−2.91 (5.16)
Update*^a^*	9.49 (12.04)

Note that estimation errors and update (the final two rows) are the variables used to compute the information integration parameters (α_G_ and α_B_) for each participant.

*^a^*Significant effect of valence (*p* < 0.05), tested using a one-sample *t* test on the mean bias scores (difference between good and bad news) for each participant.

To test for a relationship between anxiety and the asymmetry within the firefighters (i.e., preferential updating for bad news over good news), we calculated an information integration bias score for each participant. This is simply the difference between α_G_ and α_B_. A score of 0 indicates no bias in information integration in either direction, whereas positive scores indicate greater information integration for good news relative to bad news and negative scores the opposite. We then examined whether the information integration bias related to self-reported anxiety as follows: information integration bias score = β0 + β1 * self-reported anxiety + β2 * mean initial estimate + β3 * mean prior experience bias score + β4 * number of trials bias score.

Next, we ran a GLM for each of the two sets of information integration parameters (α_G_ and α_B_) separately. To ensure effects were valence specific rather than reflecting general changes in information integration, good news (α_G_) was also added as a covariate when examining information integration parameters for bad news (α_B_) and vice versa when examining information integration for good news.

For the bad news information integration parameter (α_B_), the formula for the regression in full is as follows: α_B_ = β0 + β1 * self-reported anxiety + β2 * mean initial estimate + β3 * mean prior experience bad news rating + β4 * number of bad news trials + β5 * α_G_.

For the good news information integration parameter (α_G_), the formula for the regression in full therefore is as follows: α_G_ = β0 + β1 * self-reported anxiety + β2 * mean initial estimate + β3 * mean prior experience good news rating + β4 * number of good news trials + β5 * α_B_.

Finally, we reran the analysis above, this time controlling for within-subject covariates at the within-subject level and between-subject factors at the between-subject level. Specifically, for each participant we computed an alternative set of information integration parameters, one for good news (α_G_partial_) and one for bad news (α_B_partial_), by carrying out a series of partial correlations in which absolute estimation error and update were the two variables of interest. Within-subject covariates (first estimates) were controlled for on a trial-by-trial basis (note that it was not possible to control for past experience on a trial-by-trial basis here because participants in this study completed ratings only for a subset of events). We then examined whether these alternative information integration parameters for bad news (α_B_partial_) related to self-reported anxiety, controlling for additional between-subject covariates (number of bad news trials and information integration for good news) at the between-subject level. This was done by entering alternative information integration parameters for bad news (α_B_partial_) as the dependent variable into a GLM as follows: α_B_partial_ = β0 + β1 * self-reported anxiety + β2 * number of bad news trials + β3 * α_G_partial_.

We then examined the significance of the regression weight for self-reported anxiety.

We ran the same analysis for information integration parameters for good news (α_G_partial_) as follows: α_G_partial_ = β0 + β1 * self-reported anxiety + β2 * number of good news trials + β3 * α_B_partial_.

To visualize the effect of each of these (see Fig. 5), we generated two partial regression plots. These are scatterplots of the residuals of the dependent variable (α_B_partial_ or α_G_partial_) and the independent variable of interest (self-reported anxiety) when these are regressed on the rest of the independent variables (number of bad news trials and α_G_partial_ when examining α_B_partial_, number of good news trials and α_B_partial_ when examining α_G_partial_).

## Results

### Experiment I

#### Threat manipulation was successful

**Figure 2.**
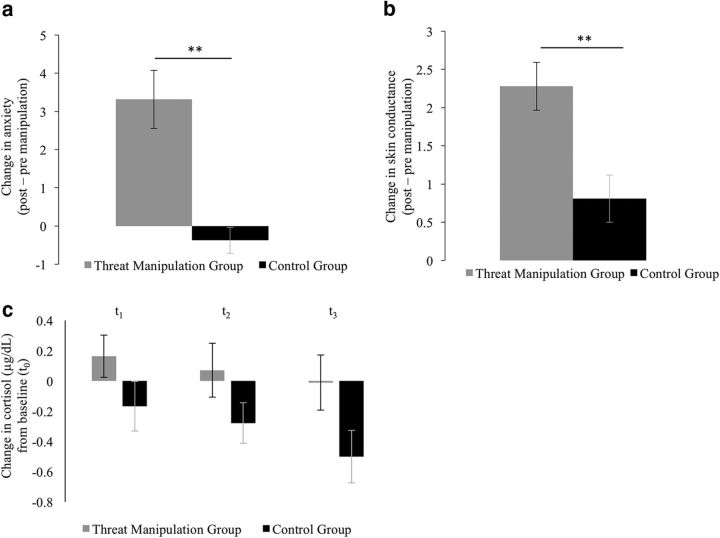
Manipulation check. Measures of self-reported state anxiety (***a***), skin conductance (***b***), and cortisol levels (***c***) were greater after manipulation relative to before in the threat manipulation group compared with the control group. Time points for cortisol measurements are as follows: t0, before threat/control manipulation procedure; t1, immediately after threat/control manipulation procedure, before undertaking the task (+10 min from t0); t2, halfway through the task (+30 min from t0); t3, after completion of task and postexperiment questionnaires (+1 h from t0). **p < 0.01 independent/paired sample ttest as appropriate; error bars represent SEM.

Subjective self-reports of anxiety and physiological measures of SCL and cortisol showed that the manipulation was effective. Specifically, after the manipulation, self-report anxiety (Fig. 2*a*) and SCL (Fig. 2*b*) showed an increase relative to before (baseline), which was greater in the threat manipulation group relative to controls (self-reported anxiety: *t*_(33)_ = 4.16, *p* < 0.001; SCL: *t*_(33)_ = 3.32, *p* = 0.002; independent-sample *t* test). There were no baseline (t0) differences in cortisol levels between the two groups (*t*_(25)_ = −0.89, *p* = 0.38). Mean cortisol levels (averaged across t1, t2, and t3) relative to baseline (t0) showed a trend toward being higher in the threat manipulation group relative to controls (*t*_(25)_ = 1.90, *p* = 0.07). This effect was driven by a reduction in cortisol levels over time in the control group (main effect of time at t1, t2, and t3 relative to baseline: *F*_(2,26)_ = 17.19, *p* < 0.001, repeated-measures ANOVA), an effect previously observed when participants become familiar with a novel experiment context (Stones et al., 1999), but an absence of this common reduction in the threat manipulation group (main effect of time: *F*_(2,22)_ = 1.00, *p* > 0.25; Fig. 2*c*). Across participants, these measures were correlated with each other (self-report and SCL: *r*_(33)_ = 0.39, *p* = 0.02; SCL and cortisol: *r*_(25)_ = 0.47, *p* = 0.01; trend for cortisol and self-report: *r*_(25)_ = 0.33, *p* = 0.09).

#### Threat eliminates asymmetric information integration

Our results show that the acute threat manipulation eliminated the well established asymmetry in information integration (Sharot et al., 2011; Moutsiana et al., 2013; Garrett et al., 2014). Specifically, the two sets of information integration parameters (α_G,_ α_B_) were entered into a group (control/threat) by valence (good news/bad news) ANOVA controlling for possible confounds (see Materials and Methods). The analysis revealed a group * valence interaction (*F*_(1,27)_ = 7.56, *p* = 0.01, η_p_^2^ = 0.22), which also remained if estimation errors were controlled for (*F*_(1,26)_ = 7.88, *p* = 0.01; Garrett and Sharot, 2017) and if the difference between number of good and bad news trials are controlled for (*F*_(1,26)_ = 6.97, *p* = 0.01).

**Figure 3.**
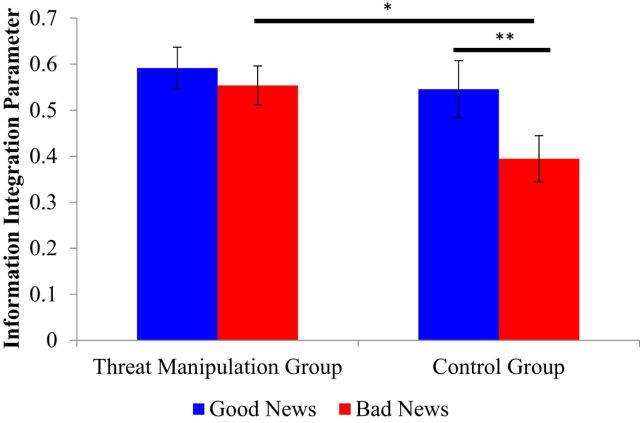
Bias in information integration parameters vanishes under threat manipulation. Whereas the control group showed asymmetrical information integration parameters (α) in response to good and bad news, this bias vanished in the threat manipulation group because of an increase in α_B_ (information integration parameter for bad news). The group * valence interaction was significant, controlling for all covariates identified in Table 1 (see Materials and Methods). *p < 0.05 independent/paired sample *t* test as appropriate; **p < 0.01 independent/paired-sample *t* test as appropriate; error bars represent SEM.

*Post hoc* tests revealed that the group * valence interaction was the result of asymmetric information integration in the control group, such that the information integration parameter was larger for good news than bad news (*t*_(15)_ = 3.34, *p* = 0.004, paired sample *t* test) but absent in the threat manipulation group (*t*_(18)_ = 0.92, *p* > 0.25, paired sample *t* test; Fig. 3). Participants in the threat manipulation group were more likely to effectively integrate bad news into their beliefs relative to those in the control group (significant difference in bad news information integration parameters α_B_: *t*_(33)_ = 2.44, *p* = 0.02, independent sample *t* test), while information integration parameters for good news (α_G_) did not differ between groups (*t*_(33)_ = 0.611, *p* > 0.250, independent sample *t* test). There were no floor or ceiling effects for α_G_ and α_B_ in the threat manipulation or control groups (all at *p* < 0.001, one-sample *t* tests vs 0 and 1, respectively), and participants' first estimates were not significantly different from the information provided (*t*_(34)_ = −0.45, *p* = 0.65, one-sample *t* test vs 0 on the difference between participants' first estimates and the information provided).

Past studies show that asymmetric information integration in this task is not associated with an asymmetry in memory (Sharot et al., 2011, 2012a,b; Moutsiana et al., 2013). In fact, asymmetry in information integration is observed even when the second estimate is elicited immediately after information is on screen (Kuzmanovic et al., 2015, 2016; Kuzmanovic and Rigoux, 2017). Here, we submitted memory scores to a group (threat manipulation/control) by valence (good news/bad news) ANOVA (see Materials and Methods for details). This did not reveal a main effect of valence (*F*_(1,33)_ = 1.24, *p* > 0.25), a main effect of group (*F*_(1,33)_ = 1.03, *p* > 0.25), or an interaction (*F*_(1,33)_ = 0.62, *p* > 0.25). This suggests that valence-dependent changes in information integration across groups cannot be attributed to memory or encoding/attention.

Conducting an ANOVA on participants' first estimates with valence (good/bad news) as a repeated factor and group (threat/control) as a between-participant factor revealed no main effect of group (*F*_(1,33)_ = 1.18, *p* > 0.25), the obvious main effect of valence (as trials are binned into good and bad according to first estimates: *F*_(1,33)_ = 278.08, *p* < 0.001) and a group * valence interaction (*F*_(1,33)_ = 6.71, *p* = 0.014). The interaction was characterized by the threat group providing lower first estimates than controls for stimuli that will subsequently be categorized as good news (*t*_(33)_ = −2.30, *p* = 0.028) but no significant difference for trials that will be subsequently categorized as bad news (*t*_(33)_ = 1.59, *p* = 0.123). Controlling for the difference between first estimates on good and bad news trials in the main ANOVA looking at information integration parameters did not alter the results (*F*_(1,26)_ = 5.43, *p* = 0.028).

What, therefore, could account for the selective fluctuations in information integration of bad news? To examine which of the changes to the psychological and physiological measures (SCL, cortisol level, self-report) could independently explain alterations in information integration of bad news, we ran a GLM in which information integration parameters for bad news (α_B_) were entered as the dependent variable and changes in self-report, SCL, and cortisol were the independent variables (all entered together in one regression). To ensure that effects were valance specific and could not be accounted for by general changes to information integration, information integration parameters for good news (α_G_) were added as a covariate as done before [Moutsiana et al., 2013; note that the same pattern of results pertains if we omit this covariate (self-reported anxiety: *F*_(1,17)_ = 4.75, *p* = 0.04; SCL: *F*_(1,17)_ = 8.81, *p* = 0.009)]. We also controlled for all other possible confounds (see Materials and Methods). The analysis revealed that changes in self-reported anxiety (*F*_(1,16)_ = 6.90, *p* = 0.02, *b*_i_ = 0.03, η_p_^2^ = 0.30) and change in physiological stress indicated by SCL (*F*_(1,16)_ = 4.99, *p* = 0.04, *b*_i_ = 0.05, η_p_^2^ = 0.24) explained the variance in information integration parameters for bad news, each of which remained significant if estimation errors were also controlled for (self-reported stress: *F*_(1,15)_ = 4.61, *p* = 0.048; SCL: *F*_(1,15)_ = 4.67, *p* = 0.047; Garrett and Sharot, 2017). In other words, participants who showed the greatest increase in SCL, which reflects the sympathetic component of the autonomic nervous system stress response (Bechara et al., 1996; Figner and Murphy, 2011), and self-reported anxiety were most likely to change their beliefs in proportion to the difference between their first estimates and the bad news received. Change in cortisol, which is suggested to reflect the HPA axis component of the stress response (Gunnar and Quevedo, 2007), did not relate to information integration for bad news (*F*_(1,16)_ = 0.46, *p* > 0.25, *b*_i_ = −0.04, η_p_^2^ = 0.03). The null result for cortisol may indicate either that the increase in bad news information integration is not associated specifically with cortisol level increase or a type II error. Ratings of emotional arousal, familiarity, and information integration parameters for good news (α_G_) were also significant predictors in the regression (see Table 3 for parameter estimates of covariates).

**Table 3.** Parameter estimates of covariates in Experiment I

	*b*_i_	SE	*t*	*p*	95% Confidence interval		η_p_^2^
					Lower bound	Upper bound	
Initial self-report STAI	−0.01	0.01	−1.10	0.29	−0.04	0.01	0.07
First estimates	0.01	0.01	0.89	0.39	−0.01	0.02	0.05
Vividness rating	−0.09	0.05	−1.84	0.09	−0.20	0.01	0.17
Familiarity rating	0.08	0.04	2.16	0.05	0.00	0.16	0.23
Prior experience rating	−0.04	0.06	−0.76	0.46	−0.17	0.08	0.04
Emotional arousal rating	−0.13	0.04	−3.03	0.01	−0.22	−0.04	0.37
Information integration parameter, good news (α_G_)	0.39	0.15	2.60	0.02	0.07	0.71	0.30

First estimates (i.e., mean initial estimations), mean ratings on subjective scales (vividness, familiarity, past experience, and emotional arousal), and α_G_ (information integration parameters for good news) were entered as covariates to account for fluctuations in α_B_ (information integration parameters for bad news). STAI, Spielberger State Trait Anxiety Inventory.

For completeness, we repeated the analysis on information integration parameters for good news, α_G_ (including information integration parameters for bad news, α_B_, and all possible covariates mentioned above), and found no significant effects (change in self-report: *F*_(1,16)_ = 0.47, *p* > 0.25, *b*_i_ = −0.01; change in SCL: *F*_(1,16)_ = 0.61, *p* > 0.25, *b*_i_ = 0.03; change in cortisol: *F*_(1,16)_ = 0.72, *p* > 0.25, *b*_i_ = 0.07).

**Figure 4.**
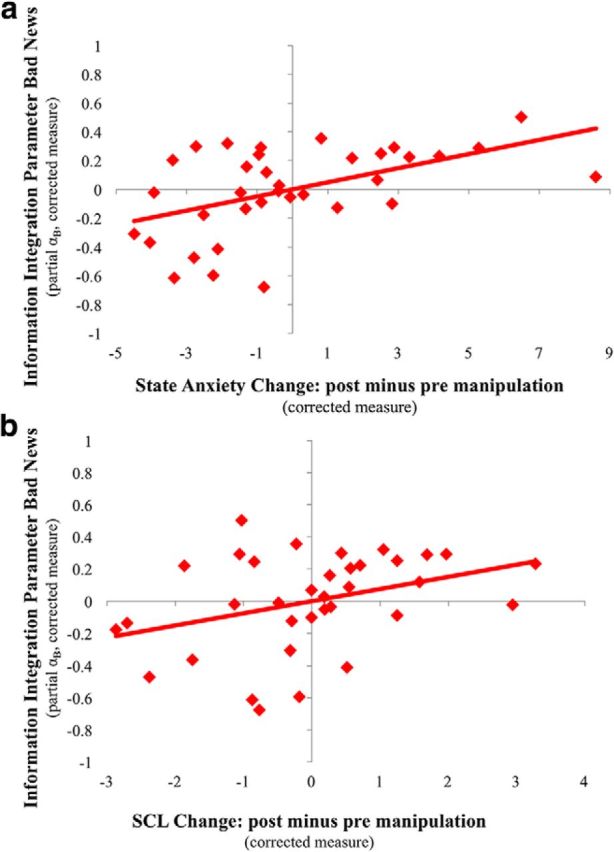
Greater integration of bad news related to self-reported anxiety and SCL. After the manipulation, an increase in both self-reported anxiety (***a***; *b*_i_ = 0.049, *p* = 0.003, η_p_^2^ = 0.25) and SCL (***b***; *b*_i_ = 0.076, *p* = .042, η_p_^2^ = 0.13) was related to larger information integration from bad news, correcting for possible confounds. Plotted are the partial regression plots from two linear models (one for self-report and one for SCL) that control for additional covariates.

Finally, we examined whether the same results are observed when controlling for within-subject covariates at the within-subject level and between-subject factors at the between-subject level. Specifically, for each participant we computed an alternative set of information integration parameters by correlating absolute estimation error and update controlling for the same within-subject covariates as above (first estimate, vividness, familiarity, past experience and emotional arousal), but controlling for them on a trial-by-trial basis. We then examined whether these alternative information integration parameters for bad news related to changes in self-reported anxiety and/or changes in SCL (additional between-subject factors, initial self-report and the alternative information integration parameters for good news, were also entered as control variables). Indeed, both effects were significant using this approach (change in self-report: *F*_(1,31)_ = 10.57, *p* = 0.003, *b*_i_ = 0.05; change in SCL: *F*_(1,31)_ = 4.51, *p* = 0.04, *b*_i_ = 0.08; Fig. 4), while the equivalent analysis on information integration parameters from good news was not (change in self-report: *F*_(1,31)_ = 0.001, *p* = > 0.25, *b*_i_ = −0.001; change in SCL: *F*_(1,31)_ = 0.55, *p* = > 0.25, *b*_i_ = 0.036).

The results of Experiment I suggested that inducing threat abolishes valence-dependent asymmetry in information integration. Thus, the previously observed bias in information integration (Sharot et al., 2011, 2012a,b; Moutsiana et al., 2013, 2015; Garrett et al., 2014; Korn et al., 2014; Kuzmanovic et al., 2015) is not constant but changes with perceived threat in the environment.

### Experiment II

Next, we set out to extend our findings from Experiment I in a natural setting. Here, we did not fashion a perceived threat but instead measured anxiety in an environment in which perceived threats would be naturally volatile. Specifically, firefighters from the state of Colorado performed the belief update task while on duty at their respective fire stations. We targeted this group of participants because they would have a naturally large range of anxiety levels owing to the volatile nature of their profession. Changes in cortisol levels were not found to be a significant predictor of information integration parameters for bad news in Experiment I. Therefore, we ruled out collecting this as a measure in Experiment II. Whereas changes in self-reported anxiety and changes in SCL were both found to be significant predictors in Experiment I, these two measures were correlated with one another (*r*_(33)_ = 0.39, *p* = 0.02). Since self-reported anxiety had the larger effect size and was easier to collect, we opted to make this our main measure.

Self-reported anxiety was significantly correlated (*r*_(26)_ = −0.51, *p* < 0.01) with the bias in information integration (i.e., α_G_ minus α_B_). In particular, heightened anxiety was associated with a reduction in the bias. This result remained significant when controlling for possible confounds (see Materials and Methods; *F*_(1,23)_ = 6.67, *p* = 0.02, η_p_^2^ = 0.23, *b*_i_ = −0.05).

To examine whether the relationship between heightened anxiety and reduced bias was the result of increased sensitivity to bad news, reduced sensitivity to good news, or both, we first constructed a GLM in which information integration parameters for bad news (α_B_) were regressed on self-reported anxiety, controlling for possible confounds (mean first estimates, mean ratings of prior experience, and number of bad news trials; see Materials and Methods for details). In addition, to ensure effects were valence specific and could not be accounted for by general changes in information integration, information integration parameters for good news (α_G_) were also added as a covariate (note, however, that the self-reported anxiety effect pertains if we omit this covariate: *F*_(1,23)_ = 9.77, *p* = 0.005). This analysis revealed that self-reported anxiety significantly explained the variance in information integration parameters for bad news, α_B_ (*F*_(1,22)_ = 10.52, *p* = 0.004, η_p_^2^ = 0.32, *b*_i_ = 0.05; Table 4), an effect that remained significant if estimation errors are also controlled for (*F*_(1,21)_ = 9.79, *p* = 0.005; Garrett and Sharot, 2017). The higher the acute anxiety reported by a firefighter, the more likely the firefighter was to integrate bad news into beliefs in proportion to the difference between their first estimations and the information provided. In this model, information integration from good news (*F*_(1,22)_ = 4.69, *p* = 0.04) was also a significant predictor of information integration from bad news. There were no floor or ceiling effects for α_G_ or α_B_ (all at *p* < 0.001, one-sample *t* tests against values of 0 and 1).

**Table 4.** Parameter estimates of covariates in Experiment II

	*b*_i_	SE	*t*	*p*	95% Confidence interval		η_p_^2^
					Lower bound	Upper bound	
First estimates	0.00	0.01	−0.03	0.97	−0.02	0.02	0.00
Prior experience rating	−0.03	0.09	−0.31	0.76	−0.21	0.15	0.00
Number of bad news trials	0.00	0.02	−0.01	0.99	−0.03	0.03	0.00
Information integration parameter, good news (α_G_)	0.34	0.16	2.17	0.04	0.02	0.67	0.18

First estimates (i.e., mean initial estimations), mean ratings of past experience, number of bad news trials, and α_G_ (information integration parameters for good news) were entered as covariates to account for fluctuations in α_B_ (information integration parameters for bad news).

We then conducted the same analysis on information integration parameters for good news (α_G_) with information integration parameters for bad news (α_B_), mean first estimates, mean ratings of prior experience, and number of good news trials as covariates. This revealed a nonsignificant trend in the opposite direction than observed for α_B_ (*F*_(1,22)_ = 3.86, *p* = 0.06, η_p_^2^ = 0.15, *b*_i_ = −0.05), such that greater self-reported anxiety was related to a trend for less information integration in response to good news. Information integration parameters for bad news (α_B_) was also significant (*F*_(1,22)_ = 7.44, *p* = 0.01, η_p_^2^ = 0.25, *b*_i_ = 0.75).

**Figure 5.**
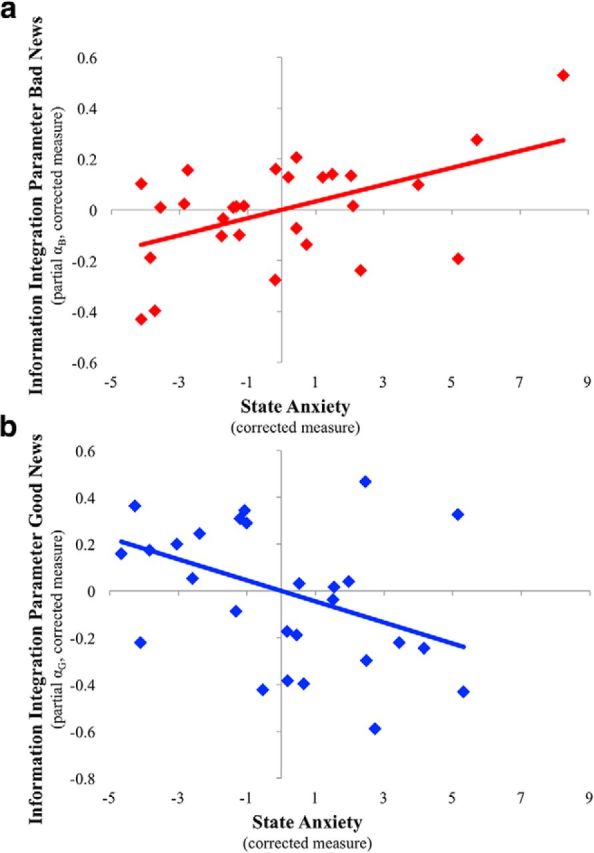
State anxiety in firefighters differentially relates to integration of good and bad news. Subjective state anxiety scores of firefighters on shift were related to larger information integration from bad news (*b*_i_ = 0.03, *p* = 0.008, η_p_^2^ = 0.26) and lower information integration from good news (*b*_i_ = −0.045, *p* = 0.038, η_p_^2^ = 0.17), correcting for possible confounds. Plotted are the partial regression plots for bad news (***a***; partial α_B_) and good news (***b***; partial α_G_) from two separate linear models (one for bad news and one for good news) that control for additional covariates.

Finally, we examined whether the same results are observed when controlling for within-subject covariates at the within-subject level and between-subject factors at the between-subject level. Under this alternative approach, higher self-reported anxiety was related to greater information integration in response to bad news (*F*_(1,24)_ = 8.34, *p* = 0.008, *b*_i_ = 0.03; Fig. 5*a*). For good news, the opposite effect was found such that higher self-reported anxiety was related to reduced information integration (*F*_(1,24)_ = 4.80, *p* = 0.038, *b*_i_ = −0.045; Fig. 5*b*). It is interesting that this latter effect was observed only in Experiment 2 and not in Experiment 1, which may indicate that natural real-life threats could have an especially strong impact on information integration processes.

These results suggest that anxiety is related to a valence-dependent enhancement in the ability to adjust beliefs in response to new information. We highlight that whereas in Experiment I threat was manipulated and thus causation could be inferred by comparing the threat manipulation and control groups, Experiment II was conducted to reveal an association in “real life”. Together, the experiments suggest that under a perceived threat (whether manipulated or naturally occurring) positively biased integration of information is not observed.

## Discussion

Our results provide evidence that the well documented asymmetry in belief formation evaporates under perceived threat. Specifically, Experiment I shows that in a low threat environment, individuals integrated information asymmetrically, faithfully incorporating good news into their existing beliefs while relatively disregarding bad news (Eil and Rao, 2011; Sharot et al., 2011). Under perceived threat, however, this asymmetry disappeared; participants showed an increased capacity to integrate bad news into prior beliefs. Increased physiological arousal and self-reported anxiety were found to correlate with enhanced integration of unfavorable information into beliefs. In Experiment II, firefighters on duty who reported higher state anxiety also exhibited greater selective integration of bad news. Because the increase in information integration in both experiments was valence specific, it cannot reflect a general improvement in learning, and because memory for the information presented was not affected, modulation of attention is an unlikely explanation.

The finding that the positivity bias in belief updating alters flexibly as a function of perceived threat reveals a potentially adaptive mechanism. In particular, the relative failure to incorporate bad news into prior beliefs leads to positively biased beliefs (also known as the optimism bias). This bias can lead to both positive effects, including increased exploration (Berger-Tal and Avgar, 2012) and motivation (Bandura, 1989), and negative effects, including failure to take precautionary action. It has been suggested that overestimating the likelihood of attaining rewards and underestimating the likelihood of harm is adaptive in environments where potential gains are sufficiently greater than costs (Johnson and Fowler, 2011). This is because under uncertainty, optimistically biased individuals will claim resources (e.g., a spouse or a job) they could not otherwise attain, as better but less optimistic competitors may walk away from the fight. Moreover, overestimating the value of novel environments can lead to an increased rate of exploration allowing the opportunity for the true value of an environment to be learned quicker (Sutton and Barto, 1998; Berger-Tal and Avgar, 2012), which is associated with superior performance in behaviors such as reproduction (Egas and Sabelis, 2001) and foraging (Rutz et al., 2006). However, in environments where potential harm is considerably greater than potential reward, computational models suggest the optimism bias to be disadvantageous (Johnson and Fowler, 2011). Thus, a valence-dependent bias in information integration that disappears under threat could be optimal in enabling a more accurate assessment of risk.

In our experiments, the source of the threat was unrelated to the information content of the task. Thus, acute stress had a valence-specific, yet general, effect on how participants used information to alter their beliefs (i.e., in response to a social threat, participants did not selectively increase their response to information about social judgment but to negative information in general). Indeed, many threat induction methods, including threat of electric shock, Cold Pressor Tasks, and the Trier Social Stress Test, produce general changes to behavior and neural responses that are not confined to the source of the threat itself (Cavanagh et al., 2011; Youssef et al., 2012; Otto et al., 2013; Robinson et al., 2013; Lenow et al., 2017). Similar findings have been observed in nonhuman animals, where different stressors have been shown to alter the degree of positive biases in a range of decision-making tasks (Harding et al., 2004; Matheson et al., 2008; Rygula et al., 2013). This may be adaptive, as threat may signify a dangerous environment that requires a general enhancement of caution.

However, if perceived threat is prolonged or dissociated from reality, enhanced integration of negative information over long periods of time could lead to psychiatric problems. We have previously shown that patients suffering from major depressive disorder (MDD) exhibit increased updating of beliefs in response to negative information relative to healthy controls (Garrett et al., 2014). MDD is often triggered by a stressful life event (Caspi et al., 2003; Roiser et al., 2012). In individuals predisposed to MDD, such a stressful life event (or series of such events) could result in prolonged periods of perceived threat and thus increased sensitivity to negative information. This in turn can form pessimistic beliefs, a symptom of MDD (Strunk et al., 2006; American Psychiatric Association, 2013), leading to even greater perceived threat about one's environment. It is possible that a similar mechanism may contribute to symptoms observed in other clinical pathologies such as in clinical anxiety and phobia.

We speculate that stress in response to perceived threat may interfere with top-down control mechanisms that may normally inhibit integration of unwanted information (for review, see Yu, 2016). A second, not mutually exclusive, possibility is that the stress reaction directly boosts the neural representation of estimation errors generated from bad, but not good, news. Indeed, it has been shown that negative prediction errors in dopamine-rich striatal nuclei are selectively amplified under threat (Robinson et al., 2013), a modulation that could be mediated by stress-induced changes to dopamine release (Schultz et al., 1997; Frank et al., 2004; Lemos et al., 2012; Sharot et al., 2012a). Future studies are required to test these hypotheses.

In summary, our results provide evidence that asymmetric information integration is not set in stone but changes acutely in response to the environment, decreasing under perceived threat. Such flexibility could be adaptive, potentially enhancing our likelihood to respond to warnings with caution in environments where future costs may be high but enabling us to maintain positive beliefs otherwise, a strategy that can, on balance, increase well being.
